# SIRT1 Activation Using CRISPR/dCas9 Promotes Regeneration of Human Corneal Endothelial Cells through Inhibiting Senescence

**DOI:** 10.3390/antiox9111085

**Published:** 2020-11-04

**Authors:** Hye Jun Joo, Dae Joong Ma, Jin Sun Hwang, Young Joo Shin

**Affiliations:** Department of Ophthalmology, Hallym University Medical Center, College of Medicine, Hallym University, Seoul 150-719, Korea; bilet12@hallym.or.kr (H.J.J.); daejoongma@hallym.or.kr (D.J.M.); hotsayme@naver.com (J.S.H.)

**Keywords:** SIRT1, corneal endothelial cells, senescence, CRISPR/dCas9

## Abstract

Human corneal endothelial cells (hCECs) are restricted in proliferative capacity in vivo. Reduction in the number of hCEC leads to persistent corneal edema requiring corneal transplantation. This study demonstrates the functions of SIRT1 in hCECs and its potential for corneal endothelial regeneration. Cell morphology, cell growth rates and proliferation-associated proteins were compared in normal and senescent hCECs. SIRT1 was activated using the CRISPR/dCas9 activation system (SIRT1a). The plasmids were transfected into CECs of six-week-old Sprague–Dawley rats using electroporation and cryoinjury was performed. Senescent cells were larger, elongated and showed lower proliferation rates and lower SIRT1 levels. SIRT1 activation promoted the wound healing of CECs. In vivo transfection of SIRT1a promoted the regeneration of CECs. The proportion of the S-phase cells was lower in senescent cells and elevated upon SIRT1a activation. SIRT1 regulated cell proliferation, proliferation-associated proteins, mitochondrial membrane potential, and oxidative stress levels. In conclusion, corneal endothelial senescence is related with a decreased SIRT1 level. SIRT1a promotes the regeneration of CECs by inhibiting cytokine-induced cell death and senescence. Gene function activation therapy using SIRT1a may serve as a novel treatment strategy for hCEC diseases.

## 1. Introduction

Cornea is the transparent window of the eye and refracts light and is maintained in a dehydrated state by corneal endothelial cells (CECs). Human CECs (hCECs) do not regenerate in vivo once they are damaged. Thus, severe damage causes corneal endothelial failure requiring corneal transplantation. However, the study of hCEC cultures has been well established [1,2]. Cultured hCECs have different characteristics depending on the donor [3,4]. Different miRNAs are expressed according to the phenotype of cultured hCECs [5]. Furthermore, an increase in cell size, variation in cell shape, and decrease in proliferative capacity have been observed in late-passage cells; this is known as senescence [6]. Senescence is the biological process of age-related deterioration of function [7]. Cells and organs gradually lose their ability to perform physiological functions and to maintain homeostasis owing to senescence [7]. Apoptotic cell death becomes more prevalent with increasing senescence in healthy fibroblast cultures [6]. The mitochondrial oxidative stress pathway is related to senescence [8]. Senescence in hCECs induces significant metabolic differences according to age [9]. In vitro observations of senescence are similar to those seen in bullous keratopathy (BK) in vivo. As they age, hCECs show a decrease in cell number, and an increase in hexagonality and cell size [4]. Therefore, elucidating the difference between healthy cultured hCECs and senescent cultured hCECs may expand our understanding of the pathogenesis of hCEC diseases.

Sirtuin 1 (SIRT1) removes acetyl groups from several transcription factors including forkhead box O1 transcription factor (Foxo1), p53, nuclear factor kappa-light-chain-enhancer of activated B cells (NF-κB), nuclear factor erythroid 2-related factor 2 (NRF2) signal transducer and activator of transcription 3 (STAT3), peroxisome proliferator-activated receptor gamma (PPARγ) and PPARγ coactivator 1-α (PGC1α) in a nicotinamide adenosine dinucleotide-dependent manner [10,11]. SIRT1 has been reported to promote proliferation and migration, and to suppress apoptosis and inflammation [12,13,14]. SIRT1 is expressed in corneal epithelial cells, keratocytes and endothelial cells [15]. SIRT1 retards aging [16] through reducing mitochondrial oxidative stress [17] and regulating energy metabolism in mitochondria [18]. Within mitochondria, SIRT1 is associated with mitochondrial DNA nucleoids and mitochondrial transcription factor A (TFAM) [19], which suggested that SIRT1 promotes mitochondrial biogenesis [20]. The pathogenesis of hCECs diseases is associated with mitochondrial dysfunction and senescence [4,21,22]. Nevertheless, the role of SIRT1 in CECs has not been investigated. Proinflammatory cytokines including tumor necrosis factor-alpha (TNF-α) and transforming growth factor-beta1 (TGF-β1) have been reported to involve the dysfunction, morphological changes and cell death of CECs [23,24,25,26]. TNF-α causes mitochondrial dysfunction by decreasing mitochondrial respiration [27], mediating mitochondrial uncoupling [28] and producing mitochondrial oxidative stress [29]. TGF-β1 inhibits mitochondrial respiration [30], stimulates reactive oxygen species (ROS) generation in mitochondria [31] and induces senescence [32]. Mitochondrial fusion and highly elongated giant mitochondria formation are observed in TGF-β-induced senescence [31].

In this study, we investigated the replicative senescence-induced changes in cultured hCECs and the effect of SIRT1 activation on cytokine-induced senescence using the CRISPR/dCas9 system.

## 2. Materials and Methods

### 2.1. Isolation and Culture of Human Corneal Endothelial Cells (hCECs)

This study was conducted in compliance with the tenets of the Declaration of Helsinki and the protocol was reviewed and approved by the ethics committee and the institutional review board of Hallym University Kangnam Hospital. hCECs were cultured according to previously described methods [33,34]. Corneas were purchased from Eversight (Ann Arbor, MI, USA), which acquired informed consent for the use of all corneas collected and cultured for the study. Corneas from six donors were used [16]. Briefly, a complex of the CEC–Descemet’s membrane was treated for 10 min in 0.25% trypsin and 0.02% ethylenediaminetetraacetic acid (EDTA) solution. After centrifugation at 1500 rpm for 3 min, cells were seeded in a FNC coating mix (Athena Environmental Sciences, Inc., Baltimore, MD, USA)-coated surfaces of six-well plates. When cells grew to be confluent (about 14–21 days), the cells was digested with 0.25% trypsin/0.02% EDTA, and passaged at the dilution of 1: 3.

hCECs were cultured and were separated into two groups based on cell size at passage 3—cells small in size (cell area < 3000 μm^2^; normal group); cells large in size (cell area > 3000 μm^2^; senescence-associated (SA) group). hCECs were compared at the same passage levels. All donor corneas were healthy. Three healthy donors were in the normal group and three were in the SA group. Ages were 25, 37, and 40 years in the normal group and 72, 75, and 67 years in the SA group. All cells showed a normal pattern at passage 0 but were sorted based on their size at passage 3.

### 2.2. Cell Transfection

To evaluate the transfection efficiency, GFP-encoded plasmids were transfected into the cells. To enhance SIRT1 expression, we used SIRT1 activation CRISPR/dCas9 system (sc-400085-ACT, Santa Cruz Biotechnology, Dallas, TX, USA). Control plasmid (sc-437275, Santa Cruz Biotechnology, Dallas, TX, USA) served as a negative control. Transfection was performed using plasmid transfection reagent (UltraCruz^®^, Santa Cruz Biotechnology Dallas, TX, USA). Cells were incubated for 72 h and collected for further experiments. SIRT1 activation was confirmed by qRT-PCR 72 h after transfection. In addition, hCECs were incubated with and without TNF-α (10 ng/mL) or TGF-β1 (10 ng/mL).

### 2.3. Cell Shape Evaluation

Cells (5 × 10^4^ cells/mL) were grown in 6-well plates for 1 week and then the cell shape was evaluated using phase-contrast microscopy. After outlining the cell borders using software (AxioVision, Rel. 4.8; Carl Zeiss Inc., Oberkocken, Germany), the size and lengths of the long and short axis of the cells was determined. The ratios of long- to short-axis length was determined as an elongation index [35]. More than 10 cells from 3 fields were employed to evaluate the cell shape.

### 2.4. Cell Viability and Proliferation Assay

Cells (5 × 10^3^ cells/well) were seeded in a 96-well plate. The cell counting kit-8 (CCK-8; Dojindo, Kumamoto, Japan) assay was utilized to determine cell viability. CCK-8 solution was added to each well and the plates were incubated for 1–2 h. The optical density was quantified at 450 nm using a microplate reader Synergy HTX (BioTek Instruments, Inc., Winooski, VT, USA). Cell viability was presented as a fold change from the control.

A bromodeoxyuridine (BrdU) incorporation assay kit (Roche Diagnostics, GmbH, Mannheim, Germany) was used for the determination of cell proliferation rate. Cells were seeded at 5 × 10^3^ cells/well in 96-well plates and incubated for 48 h at 37 °C and 5% CO_2_. BrdU was added to each well and the plates were incubated at 37 °C and 5% CO_2_. The labeling medium was removed and a FixDenat solution was added to each well. After incubation for 30 min, FixDenat solution was removed. Anti-BrdU-POD solution was added to each well and incubated for 90 min at room temperature. The cells were then washed with PBS for three times and incubated with substrate solution for 20 min at room temperature. Then, 2 N H_2_SO_4_ was used as a stopping solution. The absorbance was quantified at 450 nm using a microplate reader Synergy HTX. Proliferation rates were presented as the fold change from controls.

### 2.5. Mitochondrial Oxidative Stress Measurement

Mitochondrial superoxide levels were detected using MitoSOX^TM^ Red indicator (Invitrogen, Carlsbad, CA, USA). Cells were treated with 5 μM of MitoSOX^TM^ Red reagent (Ex/Em = 510 nm/590 nm) for 10 min at 37 °C in the dark. After washing the cells, the MitoSOX^TM^ Red fluorescence was detected. The relative fluorescence intensity was determined.

### 2.6. MitoTracker Red and Lysosome Staining

Mitochondria were stained using MitoTracker red FM fluorescent probe (Invitrogen) in accordance with the methods of the manufacturer. Cells (1 × 10^5^ cells/well) were plated in six-well plates and treated with the MitoTracker red FM at a final concentration of 200 nM for 30 min. Fluorescence images were acquired using a fluorescence microscope (DMi8, Leica, Wetzlar, Germany). Mitochondrial elongation was measured using AxioVision Rel. 4.8. software.

LysoTracker™ Green fluorescent probe (L7526, Invitrogen, Carlsbad, CA, USA) was employed to visualize lysosomes. Cells (1 × 10^6^ cells/well) grown in six-well plates were stained with LysoTracker™ Green at a final concentration of 100 nM for 30 min. Fluorescence intensity was measured using AxioVision Rel. 4.8. software (Carl Zeiss Meditec, Jena, Germany).

### 2.7. Senescence-Associated-β-Galactosidase (SA-β-gal) Assay

For senescence-associated β-galactosidase (SA-β-gal) activity, the β-Galactosidase Staining Kit (BioVision, Mountain View, CA, USA) was used. Briefly, cells were washed with PBS, fixed for 10 min using fixative solution, washed and incubated for 24 h at 37 °C with the β-galactosidase staining solution mix. Blue staining was observed under a microscope.

### 2.8. Western Blotting

Cells were lysed for 30 min in RIPA buffer supplemented with protease (Sigma-Aldrich, St. Louis, MO, USA) and phosphatase (PhosSTOP; Roche, Basel, Switzerland) inhibitor cocktails. Equal protein amounts of cell lysate were loaded on a 10% sodium dodecyl sulphate-polyacrylamide gel electrophoresis (SDS-PAGE) gel, moved to polyvinylidene difluoride membranes and blocked with 5% skim milk in PBS/0.05% Tween 20 for 1 h. Primary antibodies for GAPDH (LF-PA0212, Abfrontier, Seoul, Korea), TCF4 (TCF7L2) (sc-13027, Santa Cruz Biotechnology, Dallas, TX, USA), β-catenin (ab325572, Abcam, Cambridge, MA, USA), cyclin dependent kinase 1 (CDK1) (ab131450, Abcam, Cambridge, MA, USA), cyclin D1 (sc-718, Santa Cruz Biotechnology, Dallas, TX, USA), SIRT1 (sc-15404, Santa Cruz Biotechnology), extracellular signal-regulated protein kinases 1 and 2 (ERK1/2) (ab17942, Abcam), phospho-ERK1/2 (pERK1/2) (ab4819, Abcam), glycogen synthase kinase 3 beta (GSK3β, ab32391, Abcam), caspase-3 (sc-7148, Santa Cruz Biotechnology), notch1 (sc-376403, Santa Cruz Biotechnology), and Hes1 (sc-166410, Santa Cruz Biotechnology) were used. The immunoreactive bands were viewed using horseradish peroxidase-conjugated secondary antibodies (Bio-rad) and a WEST-Queen™ RTS Western Blot Detection Kit (iNtRON Biotechnology, Seongnam, Korea) and densitometric analysis was performed.

### 2.9. Real Time Reverse Transcription Polymerase Chain Reaction (qRT-PCR)

Total RNA was isolated from cultured hCECs using the ReliaPrep™ RNA Miniprep Systems (Promega Corporation, Madison, WI, USA) and 0.2 µg of RNA was utilized to synthesize complementary DNA (cDNA) with oligonucleotide primers using GoScript™ Reverse Transcription System (Promega Corporation, Madison, WI, USA). cDNA samples were kept at −20 °C until use. The real-time quantitative polymerase chain reaction (qRT-PCR) was conducted as previously described [36]. qRT-PCR was carried out using an AccuPower^®^ 2× Greenstar qPCR Master Mix (Bioneer, Daejeon, South Korea) and LightCycler 96 (Roche Diagnostics, Hong Kong) with the following the parameters: pre-denaturation at 95 °C for 10 min, followed by 40 cycles of denaturation (95 °C, 15 s), annealing (60 °C, 60 s), and extension (72 °C, 20 s). Primers are listed in Table 1. β-actin, was used as the housekeeping gene. Melting curve analysis was conducted for the confirmation of good-quality specific PCR products. Results were relatively quantified using the ΔΔCt method and were presented as the fold change compared to the control group. Assays were carried out in triplicate.

### 2.10. Cell Cycle Analysis

The Muse cell analyzer (Merck Millipore, Burlington, MA, USA) was used for cell-cycle analysis. Briefly, cells were cultured in 6-well plates and transfected. Cells were harvested using trypsin-EDTA solution, washed twice with PBS, fixed in 70% ethanol overnight at −20 °C and stained with 200 μL solution containing 50 μg/mL of PI and 100 μg/mL of RNase A (Biosesang, Seongnam, Korea). The data were presented as the proportion of the cells in the G0/G1 phase, S phase, and G2/M phase.

### 2.11. Mitochondrial Membrane Potential

Muse™ MitoPotential assay (Merck Millipore, Guyancourt, France) was used for evaluating mitochondrial membrane potential. MitoPotential Dye, which is a lipophilic cationic probe to determine the alterations in the mitochondrial membrane potential, and 7-AAD, which is a marker of cell death, were used. The data were obtained using the Muse™ Cell Analyzer (Guyancourt, France).

### 2.12. Animal Study and In Vivo Transfection

Six-week-old Sprague–Dawley (SD) rats were involved in this study. Five rats were involved in each group. The rats were housed in a standard 12:12 h light-dark cycle at 25 °C for 7 days before initiating the experiments. All studies involving animals were conducted according to the Association for Research in Vision and Ophthalmology (ARVO) Statement for the Use of Animals in Ophthalmic and Vision Research and were approved by the Institutional Animal Care and Use Committees (IACUC) of Hallym University Medical Center.

For in vivo gene delivery, 0.1 nmol of negative control plasmid (sc-437275, Santa Cruz Biotechnology; Control) or plasmid for SIRT1 activation CRISPR/dCAS9 system (sc-400085-ACT, Santa Cruz Biotechnology; SIRT1a) was injected intracamerally in the SD rats. Then, electroporation was performed with 7 mm Tweezertrodes (BTX, Holliston, MA, USA). The pulses for electroporation were set at 140 V, 950 milliseconds interval, five pulses, and 100 milliseconds length. Cryoinjury was performed by placing it in touch with a 3 mm-diameter metal rod for 10 s chilled in LN2 for 10 min two days after electroporation, as previously published [36]. Then, corneas were flushed with normal saline (day 0).

### 2.13. Clinical Evaluation and Alizarin Red S Staining

Corneal opacity was graded with photographs on days 0, 3, 5, 7, 9, 11 and 14. Corneal opacity was graded as previously established [37]. After enucleating the eyes, the eyes were fixed in 3.7% formaldehyde. The corneal buttons were removed, and then immunofluorescence staining for SIRT1 was performed. The corneas were incubated overnight in rabbit anti-human SIRT1 antibody (sc-15404, Santa Cruz Biotechnology), washed with PBS and incubated with secondary fluorescein isothiocyanate (FITC)-conjugated anti-rabbit IgG antibody. Hoechst 33,342 was used for nuclear staining. Vital staining was performed with 0.2% alizarin red S in 0.9% NaCl (pH 4.2) for 90 s as previously described [37], followed by fixation with 2.5% glutaraldehyde. Then, the corneas were excised, and mounted on the slides under a coverslip with one drop of 0.9% NaCl. The corneal endothelium was observed under a microscope (DM2000; Leica, Wetzlar, Germany), and images were taken. Cells at the central area were counted at 400× magnification.

### 2.14. Statistics

All data were presented as the mean ± standard deviation. Graphpad Prism 8.0 (GraphPad Software Inc., La Jolla, CA, USA) was employed for all statistical analysis. An independent *t*-test was used to compare the differences between two groups and the one-way analysis of variance (ANOVA) was employed to compare the mean differences across 3 groups. The p value of less than 0.05 was regarded as a significantly statistical difference. Experiments were performed in triplicate, and a representative experiment is shown.

## 3. Results

### 3.1. Senescence of Human Corneal Endothelial Cells

#### 3.1.1. Cell Shape, Viability, Senescence, Proliferation, and Cell Cycle Analyses

**Figure 1:**
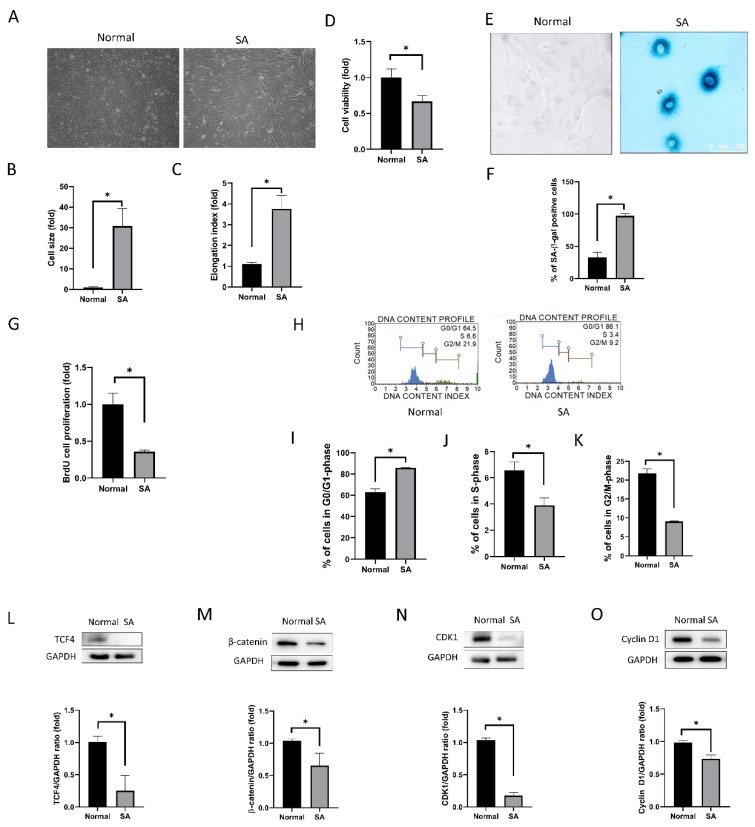
Cell shape, senescence-associated (SA)-β–gal assay, proliferation, and cell cycle. (**A**) Inverted microscopy image showing cell shape in normal and SA groups. (**B**) Comparison of cell size between normal and SA groups. (**C**) Elongation index for normal and SA groups is shown. (**D**) Comparison of cell viability between normal and SA groups. (**E**,**F**) Evaluation of senescence in normal and SA groups. The proportion of SA-β–gal stained cells was higher in the SA group. (**G**) Cell proliferation rate was measured by bromodeoxyuridine (BrdU) incorporation assay. (**H**) Cell cycle analysis was performed. (**I**–**K**) The proportion of cells in the G0/G1, S and G2/M phase are shown. (**L**) TCF4 expression was decreased in the SA group. (**M**) β-catenin expression was decreased in the SA group. (**N**) Cyclin dependent kinase 1 (CDK1) expression was decreased in the SA group. (**O**) Cyclin D1 expression was not different between the normal and SA groups. All experiments were conducted in triplicate or quadruplicate. * for *p* < 0.05 using independent *t*-test.

Cells were sorted into two groups based on cell size at passage 3, normal group with cell area < 3000 μm2 and SA group with cell area > 3000 μm^2^. Cell shape and size were different between the normal and SA cells (Figure 1A). Cell size was larger in the SA group than in the normal group (*p* < 0.001, independent *t*-test; Figure 1B). The elongation factor was 1.10 ± 0.09 for the normal group and 3.77 ± 0.65 for the SA group (*p* < 0.001; Figure 1C). Cell viability was lower in the SA group than in the normal group (*p* < 0.001; Figure 1D). SA-β-gal staining was performed (Figure 1E). SA-β-gal has been widely employed as a marker of senescence since senescent cells alter the activity of the lysosomal β-gal [38]. The proportion of SA-β-gal stained cells was higher in the SA group (97.1 ± 3.4) than in the normal group (32.2 ± 8.2) (*p* < 0.001, using independent *t*-test) (Figure 1F).

The cell proliferation rate, as determined by BrdU incorporation assay, was lower in the SA group than in the normal group (*p* < 0.001; Figure 1G). Results of cell cycle analysis were different for the normal group and SA group (Figure 1H). Cells from the SA group were arrested in the G0/G1 phase. The proportion of cells in the G0/G1 phase was higher in the SA group than in the normal group (*p* < 0.001, using independent *t*-test; Figure 1I). The proportion of cells in the S-phase and in the G2/M phase was significantly lower in the SA group than in the normal group (*p* < 0.001 for all data points, using independent *t*-test; Figure 1J,K).

The expression of proliferation-associated proteins was different between the normal group and the SA group. TCF4 (TCF7L2) expression levels were lower in the SA group than in the normal group (*p* = 0.006, using independent *t*-test; Figure 1L). β-catenin expression levels were lower in the SA group than in the normal group (*p* = 0.003; Figure 1M). CDK1 expression levels, which are associated with the progression of the cell cycle, were lower in the SA group compared to the normal group (*p* = 0.005; Figure 1N). Cyclin D1 expression levels were lower in the SA group compared to the normal group (*p* = 0.026; Figure 1O).

#### 3.1.2. Mitochondrial Oxidative Stress, MitoTracker Red Staining, and Lysosome Staining

**Figure 2:**
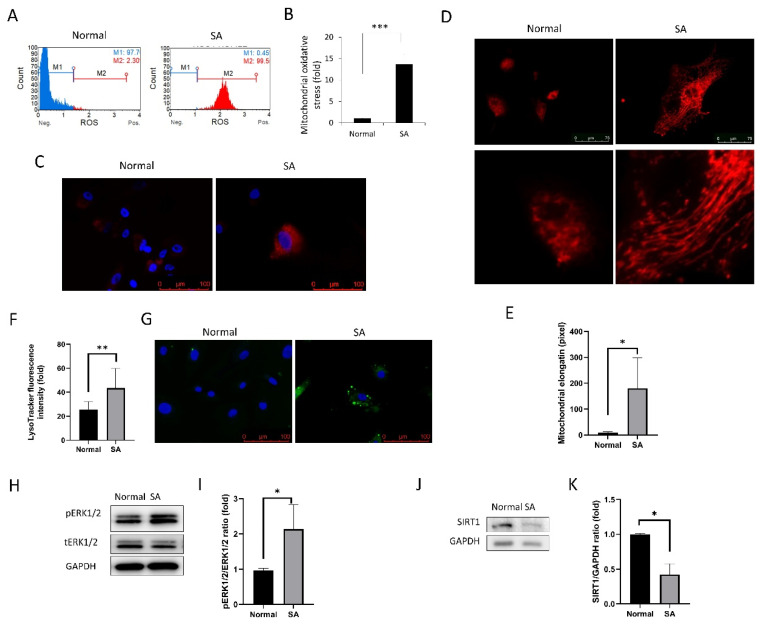
Mitochondrial oxidative stress, elongation, and lysosome staining. (**A**) MitoSOX staining intensity of cells as analyzed using the Muse cell analyzer. (**B**) Mitochondrial oxidative stress levels were compared between the normal and SA groups. (**C**) Fluorescence imaging using the MitoSOX probe shows mitochondrial oxidative stress in cells. (**D**) MitoTracker red was used for mitochondrial imaging of cells from normal and SA groups. Mitochondrial elongation is shown in the SA group. (**E**) Mitochondrial elongation is greater in the SA group as compared to that in the normal group. (**F**,**G**) Lysosomal staining of cells from the normal and SA groups. (**H**,**I**) phospho- extracellular signal-regulated protein kinases 1 and 2 (pERK1/2) expression levels are shown. (**J**,**K**) SIRT1 expression levels are shown. All experiments were performed in triplicate or quadruplicate. * for *p* < 0.05 using independent *t*-test.

Mitochondrial oxidative stress levels, as measured by MitoSOX^TM^ Red staining (Invitrogen), increased in the SA group compared to the normal group (*p* < 0.001; Figure 2A–C). MitoTracker Red fluorescence was used for evaluating mitochondrial elongation [39]. Mitochondrial elongation was greater in the SA group than in the normal group (*p* < 0.001; Figure 2D,E). LysoTracker™ Green (L7526, Invitrogen) was used to visualize the lysosomes which were prominently visible in senescent cells (Figure 2F,G). The expression levels of phospho- pERK1/2 were elevated in the SA group compared to the normal group (*p* = 0.043; Figure 2H,I). Moreover, the expression levels of SIRT1 were reduced in the SA group compared to the normal group (*p* = 0.003; Figure 2J,K).

#### 3.1.3. Animal Study of SIRT1 Activation Using CRISPR/dCas9 in Rat Corneal Endothelial Cells

**Figure 3:**
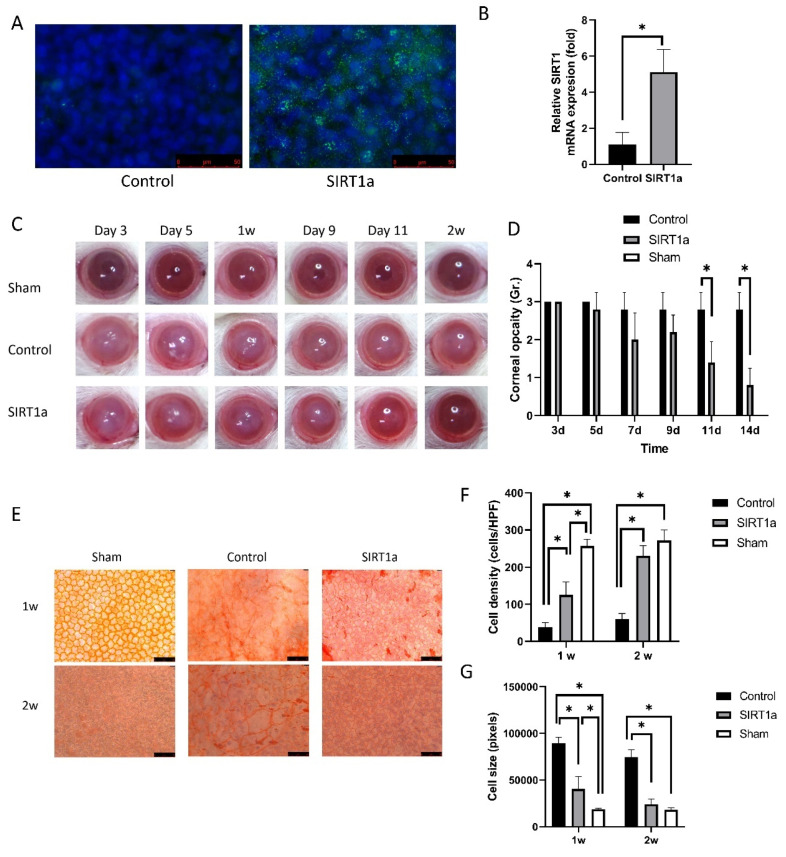
Animal study of SIRT1 activation using CRISPR/dCas9 in rat corneal endothelial cells (CECs). (**A**) Immunofluorescence staining of SIRT1 showing SIRT1a overexpression in SIRT1a group. (**B**) Real-time quantitative polymerase chain reaction (qRT-PCR) showing that relative SIRT1 mRNA expression was elevated to 246.7% of the control group. (**C**,**D**) Corneal opacity in SIRT1a group was decreased compared to control group on days 11 and 14. (**E**–**G**) Alizarin red S staining showed higher density of CECs at the center in the SIRT1a group compared to the control group, as well as smaller cell size in the SIRT1a group compared to the control group. All experiments were performed in triplicate or quadruplicate. * for *p* < 0.05 using independent *t*-test.

Immunofluorescence staining of SIRT1 showed SIRT1a overexpression in SIRT1a group (Figure 3A). qRT-PCR showed that relative SIRT1 mRNA level was elevated to 246.7 ± 12.5% of the control group (*p* = 0.008, Figure 3B). Corneal opacity was different among groups (*p* < 0.001, ANOVA). Corneal opacity in the SIRT1a group was decreased compared to the control group on days 11 and 14 (*p* < 0.001 for both; Figure 3C,D). Alizarin red S staining showed that the density of CECs at the center and the cell size was different among groups (*p* < 0.001 for all, ANOVA). The densities of CECs at the center were higher in the SIRT1a group than in the control group and the cell size was smaller in the SIRT1a group compared to the control group (*p* < 0.001 for all; Figure 3E–G). The densities and sizes of CECs in SIRT1a group were lower and larger compared to Sham at 1 week (*p* < 0.001 for both). Then, there was no difference in the densities and sizes of CECs between SIRT1a group and Sham at 2 weeks.

#### 3.1.4. SIRT1 Activation Using CRISPR/dCas9 in Cultured Human Corneal Endothelial Cells

**Figure 4:**
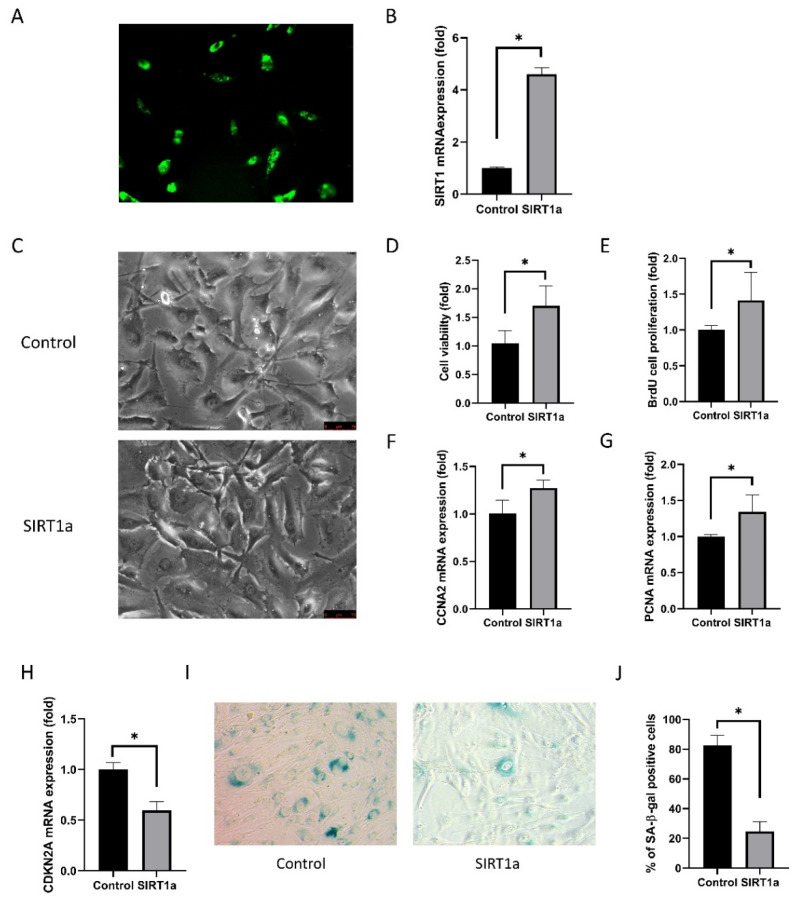
SIRT1 activation using CRISPR/dCas9 in cultured human corneal endothelial cells. (**A**) GFP-encoded plasmids were transfected into the cells. Green indicates GFP. (**B**) Relative SIRT1 mRNA expression in cultured hCECs of SIRT1a group was elevated to 247% of control. (**C**) Cell shape was polygonal in the SIRT1a group compared to the control. (**D**, **E**) Cell viability and proliferation rate were higher in SIRT1a group compared to control. (**F**, **G**) Relative mRNA expression levels of CCNA2 and PCNA were increased in SIRT1a group compared to control. (**H**) CDKN2A mRNA expression was lower in SIRT1a group compared to control. (**I**, **J**) The number of SA-β-gal positive cells were lower in SIRT1a group compared to control. All experiments were performed in triplicate or quadruplicate. * for *p* < 0.05 using independent *t*-test.

GFP-encoded plasmids were transfected into the cells (Figure 4A). The transfection efficiency was 92.1 ± 2.0%. Relative SIRT1 mRNA expression in cultured hCECs of the SIRT1a group was elevated to 247% of the control (*p* < 0.001; Figure 4B). Cell shape was polygonal in the SIRT1a group compared to the control (Figure 4C). Cell viability and proliferation rate was higher in the SIRT1a group compared to the control (*p* = 0.004 and 0.048; Figure 4D,E). Relative mRNA expression levels of CCNA2 and PCNA were increased in the SIRT1a group compared to the control (*p* = 0.008 and 0.028; Figure 4F,G). CDKN2A mRNA expression was lower in the SIRT1a group compared to the control (*p* < 0.001; Figure 4H). The proportion of SA-β-gal stained cells was lower in the SIRT1a group than in the control group (*p* < 0.001; Figure 4I,J).

#### 3.1.5. SIRT1 Inhibits Cytokine-Induced Cell Death

**Figure 5:**
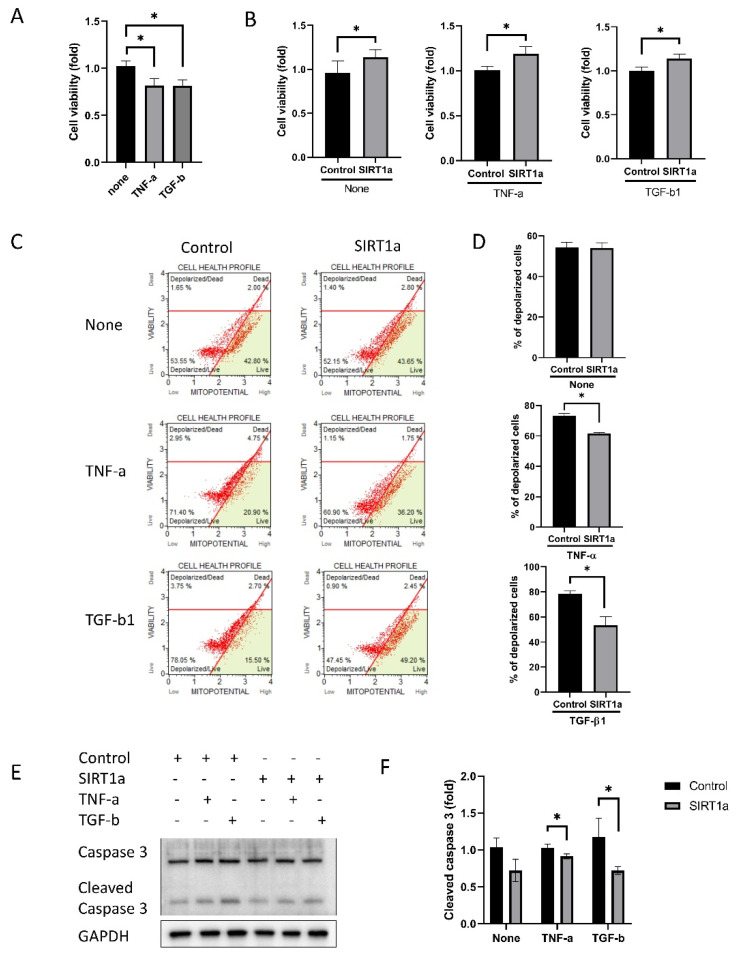
SIRT1 inhibits cytokine-induced cell death. (**A**) Cells were treated with tumor necrosis factor-alpha (TNF-α) and transforming growth factor-beta1 (TGF-β1). Cell viability was reduced when treated with TNF-α or TGF-β1. (**B**) Cell viability was increased in SIRT1a group compared to control in untreated or upon treatment with either TNF-α or TGF-β1. (**C**,**D**) Mitochondrial membrane potential was depolarized upon TNF-α or TGF-β1 treatment. The number of depolarized cells was increased in SIRT1a group compared to control upon treatment with TNF-α or TGF-β1. (**E**,**F**) Cleaved caspase 3 level was decreased in SIRT1a group compared to control upon treatment with TNF-α or TGF-β1. All experiments were performed in triplicate or quadruplicate * for *p* < 0.05 using independent *t*-test.

Cell viability was reduced in cells treated with TNF-α or TGF-β1 (*p* = 0.004 for both; Figure 5A). Cell viability was increased in the SIRT1a group compared to the control both when untreated or when treated with either TNF-α or TGF-β1 (*p* = 0.028, < 0.001 and < 0.001; Figure 5B). Mitochondrial membrane potential was depolarized upon TNF-α or TGF-β1 treatment (Figure 5C). The proportion of depolarized cells was elevated in the SIRT1a group compared to the control upon treatment with TNF-α or TGF-β1 (*p* < 0.001 for both; Figure 5C,D). The level cleaved caspase 3 was decreased in SIRT1a group compared to control upon treatment with TNF-α or TGF-β1 (*p* = 0.028 and 0.038; Figure 5E,F).

#### 3.1.6. SIRT1 Inhibits Cytokine-Induced Cell Cycle Arrest

**Figure 6:**
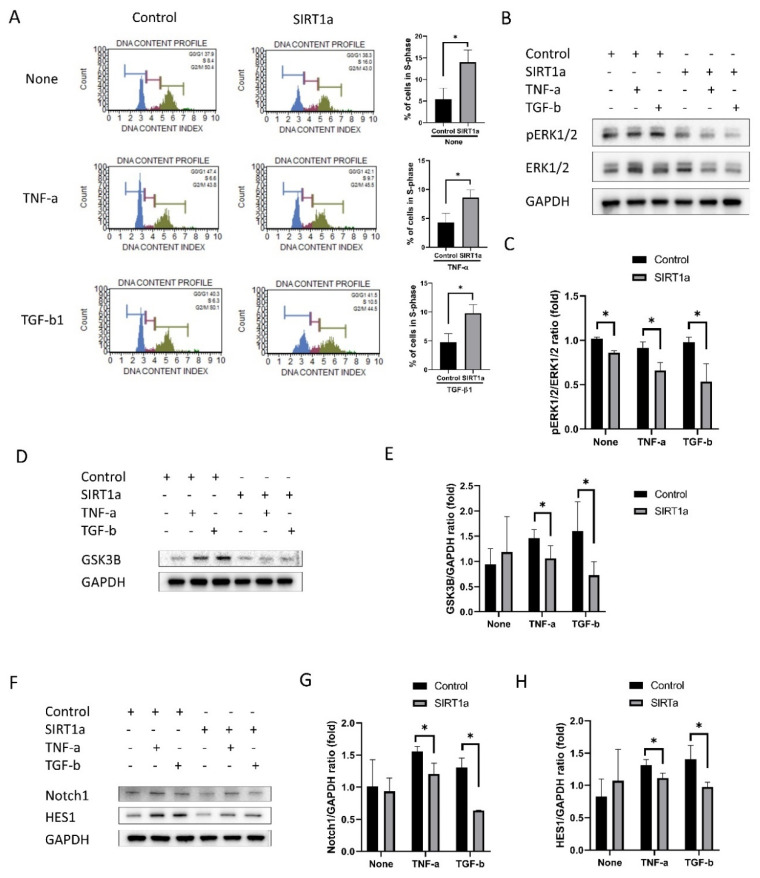
SIRT1 inhibits cytokine-induced cell cycle arrest. (**A**) Cell cycle analysis showed that the number of cells in S-phase was increased in SIRT1a group compared to control in untreated and upon treatment with either tumor necrosis factor-alpha (TNF-α) and transforming growth factor-beta1 (TGF-β1). (**B**–**H**) phospho- extracellular signal-regulated protein kinases 1 and 2, glycogen synthase kinase 3 beta (GSK3β), Notch and HES1 levels were reduced in SIRT1a group compared to control upon treatment with TNF-α or TGF-β1. All experiments were performed in triplicate or quadruplicate. * for *p* < 0.05 using independent *t*-test.

Cell cycle analysis by DNA content measurement revealed that the proportion of cells in S-phase was elevated in SIRT1a group compared to the control in untreated or upon treatment with either TNF-α or TGF-β1 (*p* = 0.040, 0.012 and 0.007; Figure 6A). pERK1/2, GSK3β, Notch1 and HES1 levels were reduced in the SIRT1a group compared to the control upon treatment with TNF-α or TGF-β1 (*p* = 0.018 and 0.021 for pERK1/2, *p* = 0.039 and 0.035 for GSK3β, *p* = 0.029 and 0.009 for Notch1, and *p* = 0.036 and 0.032 for HES1; Figure 6B–H).

## 4. Discussion

Senescent cells do not proliferate in response to mitogenic signals, which induces the failure of regeneration in wound healing [40]. Senescence is one of the causes of hCEC diseases in which the number of CECs is reduced and corneal grafting is required to treat the condition [41]. SA group consisted of cells showing senescence characteristics, which were large-sized, did not proliferate at passage 3 and were donated from old-aged donors, different from normal group. In this study, we compared hCECs in the SA group and normal hCECs. The SA group showed a greater proportion of SA-β-gal stained cells, which is a marker of senescence, as well as greater elongation index and lower cell viability. Senescence is accompanied by characteristic morphological changes, including cell elongation, increased cell size, nuclear size, and increased number of vacuoles in the cytoplasm [42,43], because it affects cytoskeletal turnover and disruption [44,45]. In this study, cells in the SA group showed the G0/G1 phase arrest of the cell cycle, lower proliferation rates and a corresponding decrease in relevant protein expression. G1 phase arrest of cell cycle, which is a common feature of senescence [46], is similar to the phenomenon observed in hCECs in vivo [47,48]. Senescent cells are in a state of permanent cessation of proliferation [49].

Senescent cells have higher levels of oxidative stress [49], which are produced in mitochondria [50]. In this study, mitochondrial oxidative stress levels, which are associated with senescence [50], were higher in the SA group. Mitochondria serve as energy producing organelles and as a source of ROS [31]. Energy production is accompanied by a corresponding elevation in oxidative stress levels [51], which reduce the self-renewing ability of stem cells and decrease cell proliferation [52]. Mitochondrial DNA (mtDNA) is more susceptible to oxidative damage, since the levels of 8-oxo-dG, one of the indicators of common oxidative DNA lesions, are found to be higher in mtDNA than those in nuclear DNA [53]. In this study, mitochondrial elongation was also greater in the SA group. Mitochondria exist as isolated organelles floating in the cytoplasm [54], and mitochondrial biogenesis drives cell proliferation [55]. A balance between mitochondrial fission and fusion dynamically regulates mitochondrial morphology [56]. Mitochondrial fission is necessary for cell division [56], and failure of fission induces mitochondrial elongation [57], which is a characteristic of senescence and is followed by cell cycle arrest [57].

In this study, the cells in the SA group showed the presence of prominent lysosomes, which are membrane-bound organelles that contain hydrolytic enzymes that digest cellular structures or debris [58]. Lysosomes play a central role in cellular homeostasis by regulating cellular clearance and the impairment of lysosomes has been observed in aging organisms [58]. Cellular metabolites, such as iron and calcium, are widely involved in lysosome function [59]. In this study, cells in the SA group showed a reduction in SIRT1 expression and an elevation in phosphorylation of ERK1/2. Activation of ERK1/2 plays a critical role in cell survival and in the induction of senescence under cellular stress [60] and its phosphorylation was regulated by lysosomes in turn [61,62]. These results indicate that senescent cells are metabolically active, although cellular senescence is a state of permanent cessation of proliferation [49]. SIRT1 is an enzyme that deacetylates proteins and regulates age-related cellular processes through the autophagy–lysosomal pathway [63]. Our results suggest that senescence is a major mechanism participating in the onset and progression of BK and is a putative target for the novel therapy of BK.

SIRT1 is a histone deacetylase in the nucleus and cytoplasm and has multiple physiological functions [17]. It has an anti-aging effect by modulating mitochondrial gene expression, mitochondrial biogenesis, and reactive oxygen metabolism through the Forkhead box O (FoxO) transcription factor family [16,64]. We investigated the effect of SIRT1 activation on CECs using the SIRT1 activation CRISPR/dCas9 system. In vivo study showed that SIRT1a promoted wound healing in the CECs of rats after cryoinjury. The CRISPR/dCas9 system activates the target gene through specifically binding to a target DNA sequence based on sequence-specific RNA-guided system [65]. The advantage of CRISPR/dCas9 is that it induces endogenous expression of SIRT1 in situ, and not ectopic. Electroporation was performed to efficiently introduce genes into the corneal endothelium in vivo with minimal cell damage [36]. In this study, transfection of SIRT1a using electroporation was effective (Figure 3). Cryoinjury is a method often used to induce corneal endothelial injury [36]. In this study, the cryoinjury of CECs caused corneal edema and the cornea became cloudy and SIRTa reduced cryoinjury-induced corneal clouding and promoted the healing of CECs [36]. SIRT1 facilitates high glucose-delayed wound healing through p53 regulation [66]. In vitro experiments were conducted to understand the effect of SIRT1a on CECs. In this study, SIRT1 promotes the proliferation of hCECs via blocking senescence. Cell shape was a polygonal shape in the SIRT1a group. Cell viability and proliferation were not only increased in SIRT1a, but the levels of related molecules were also altered. Cyclin A2 is a crucial modulator of the cell cycle and activates cyclin-dependent kinases that function in the regulation of both S phase and mitotic entry [67]. CDKN2A negatively regulates the G1/S transition of mitotic cell cycles and promotes cellular senescence [68]. PCNA is expressed during the cell cycle and interacts with cell cycle proteins and its expression is decreased in senescence [69,70]. SA-β-gal staining is commonly employed as an indicator of senescence [71], which was suppressed by SIRT1a.

Then, we examined the effect of SIRT1a on cytokine-induced changes in hCECs. TNF-α and TGF-β1 were used as inflammatory cytokines, which induce the damage and dysfunction of mitochondria [72]. In this study, SIRT1a inhibits cytokine-induced cell cycle arrest, as revealed by cell cycle analysis, the inhibited activation of ERK1/2 and expressions of GSK3β, Notch1 and Hes1. TNF-α and TGF-β1 promote premature senescence, such as the cessation of proliferation, cell cycle arrest and an increase in CDKN2A [73]. ERK1/2, which is activated in senescence and elevates metabolic activity although cell cycle is arrested, is involved in the p38 MAPK pathway and this pathway is inhibited by SIRT1 [74]. GSK3β regulates mitochondrial energy metabolism [75] and is inactivated by SIRT1, which promotes cell proliferation [76]. SIRT1 negatively regulates Notch1 [77], which activates the arrested cell cycle and inhibits cell proliferation [78], and Hes1 transactivation by Notch1 [79].

Persistent, unregulated oxidative stress in senescent cells causes cell death. TNF-α and TGF-β1 increased ROS levels, which induces cell death [80,81]. TNF-α is a cytokine involved in systemic inflammation and exerts pleiotropic effects on various cell types [82]. TNF-α functions through the canonical NF-κB pathway, stress kinases, and the apoptotic caspase pathway [83]. TGF-β1 is associated with many cellular functions, including cell growth, senescence, cell differentiation, and apoptosis [84]. In this study, SIRT1 inhibits cytokine-induced cell death. Cell viability and mitochondrial membrane potential, which was reduced after treatment with TNF-α or TGF-β1, was increased by SIRT1a. Mitochondrial membrane potential is essential for the maintenance of cellular health and viability by energy storage during oxidative phosphorylation. The loss of mitochondrial membrane potential is followed by apoptosis, in which caspase 3 is activated as one of the central executioners [85]. SIRT1a protects the cells against apoptosis by maintaining the mitochondrial membrane potential and activating PGC-1α [86].

## 5. Conclusions

Senescence, which is one of the causal mechanisms of hCEC diseases, was associated with SIRT1 reduction. SIRT1a promotes corneal endothelial wound healing during in vivo experiments and the proliferation of CECs in vitro experiments by inhibiting cytokine-induced cell death and senescence. Gene function activation therapy using SIRT1a may serve as a novel treatment strategy for hCEC diseases.

## Figures and Tables

**Table 1:** Primer sequence for RT-qPCR.

Gene	Forward	Reverse
SIRT1 (NM_012238)	TCGCAACTATACCCAGAACATAGACA	CTGTTGCAAAGGAACCATGACA
COL8A2 (NM_005202.)	GGCAAAGGCCAGTACCTG	CCCCTCGTATTCCTGGCT
CDKN2A (NM_000077)	CATAGATGCCGCGGAAGGT	CTAAGTTTCCCGAGGTTTCTCAGA
PCNA (NM_002592)	GCGTGAACCTCACCAGTATGT	TCTTCGGCCCTTAGTGTAATGAT
CDC25B (NM_021873)	GGCTGAGGAACCTAAAGCCC	CTTTCCGTCTACTGTCTGTAGGA
ACTB (NM_001101)	AGAGCTACGCTGCCTGAC	AGCACTGTTGGCGTACAG
